# Supplementary material for: Amplification of chirality in surface-confined supramolecular bilayers
Source: Nat Commun. 2018 Aug 24;9:3416. doi: 10.1038/s41467-018-05962-3 (PMC6109073; doi:10.1038/s41467-018-05962-3)
Supplement: Supplementary file 1 — Supplementary Information [file 41467_2018_5962_MOESM1_ESM.pdf]

Supplementary information for

Amplification of chirality in surface-confined supramolecular bilayers

Cao et al

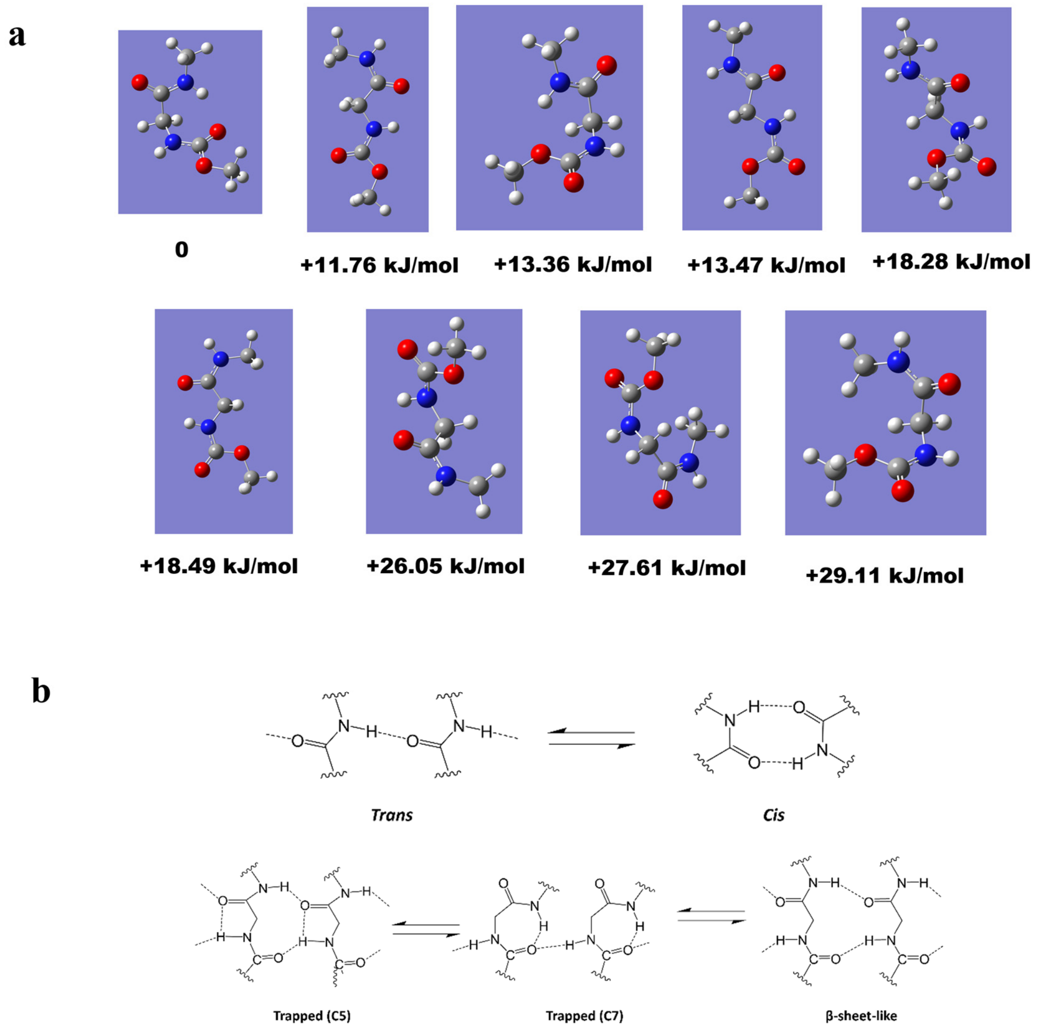

Supplementary Figure 1. Flexibility of amino acid. a, All the conceivable conformations of a glycine segment searched by DFT calculations. **b,** The *trans*- and *cis*-forms of a peptide bond. The former can form extensive chains while the latter is favoured for dimerization. **c,** Three possible ways for the formation of extensive hydrogen bonding networks between amino acid segments.

While nearly all these conformations are non-superimposable with their mirror images, the helical conformations we discussed in the main manuscript are the most stable ones. The two helical conformations of glycine are equal in energy, but a small energy difference between the two conformers of an enantiomer is induced by the methyl side chain. The barrier for helix reversal to a less preferred conformer of an enantiomer amounts to 18.5 kJ/mol, comparable to the barrier for anti-to-gauche isomerism of butane (14.2 kJ/mol), implying that that the helix reversal of an enantiomer should be very easy to occur at the monomeric state.

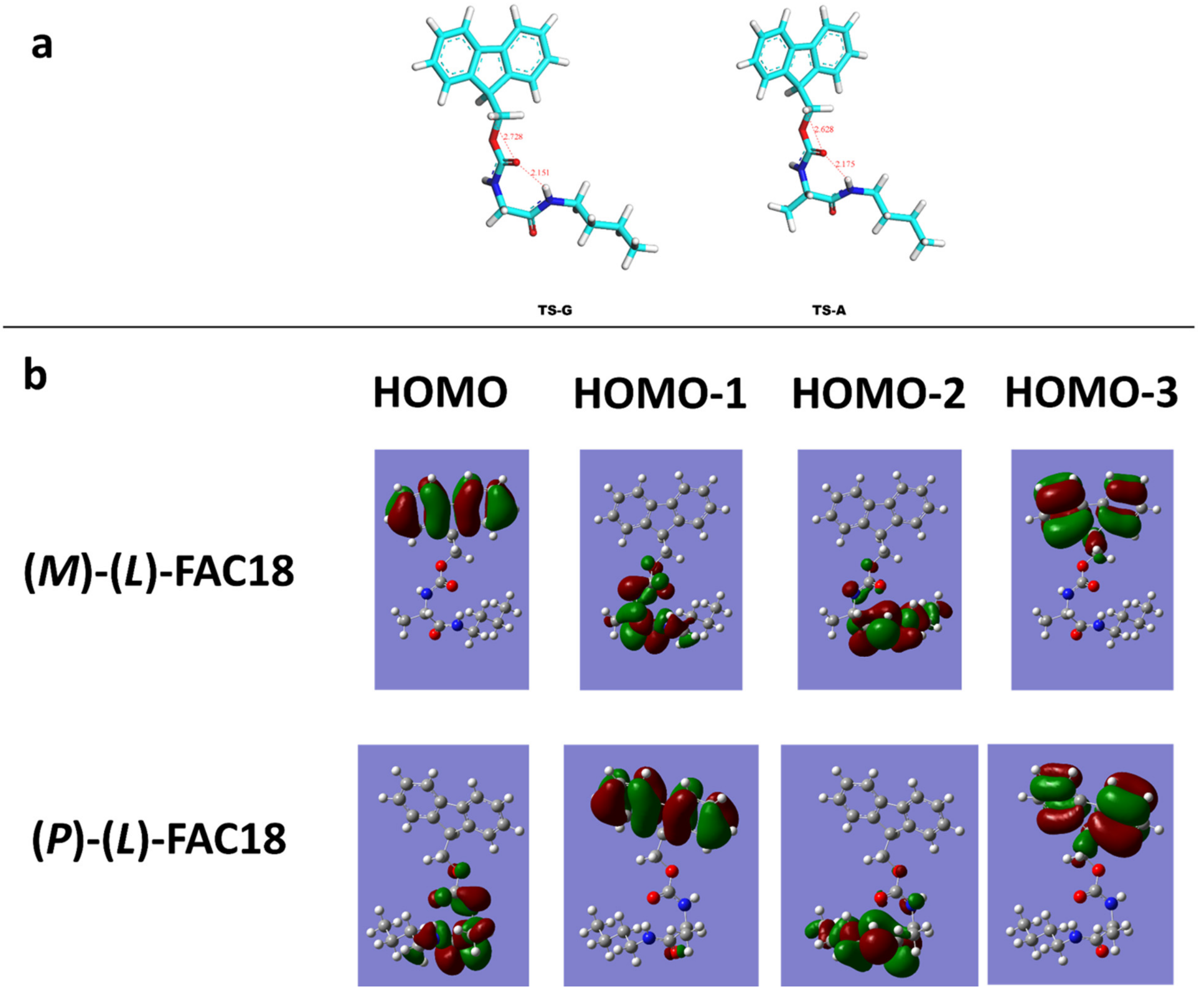

Supplementary Figure 2. Transition states and HOMO. a, Structures of the transition states in Fig. 1. **b,** HOMO to HOMO-3 of the two conformers of (*L*)-FAC18.

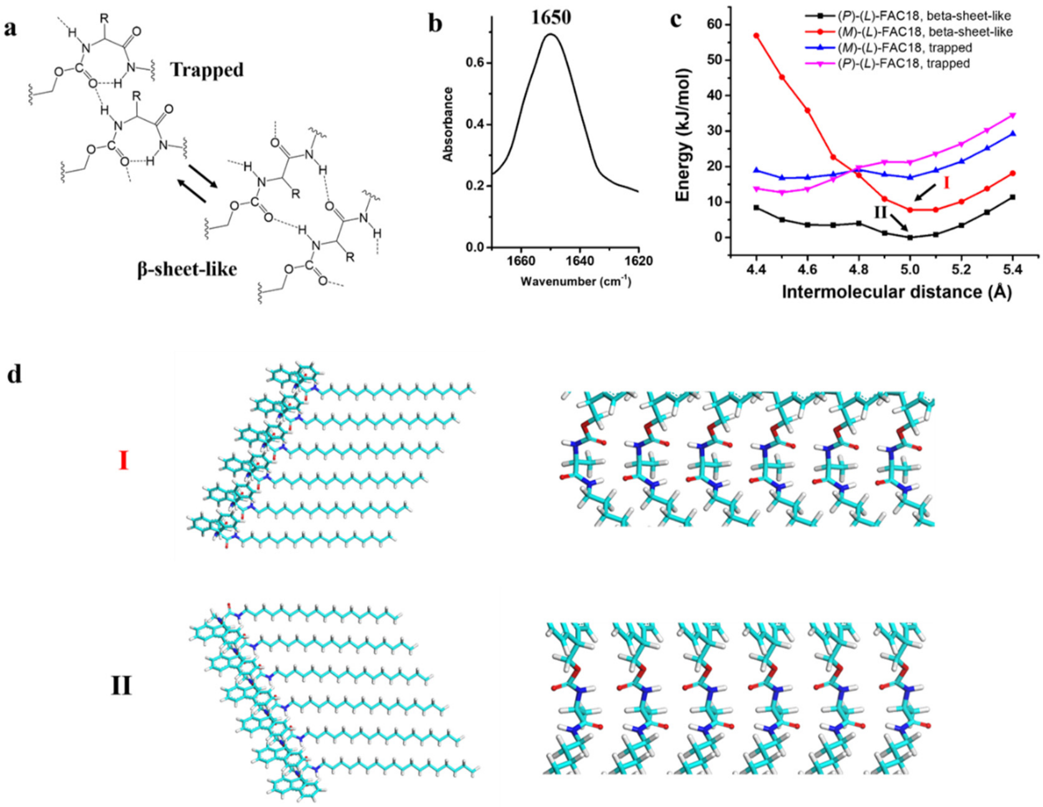

Supplementary Figure 3. Conformationally trapped and β-sheet–like arrangements of (*L*)-FAC18. a, Chemical equilibrium between a conformationally trapped and a β-sheet-like arrangement. **b**, Infrared amide I band of the nanostructures formed by (*L*)-FAC18 in hexane. **c**, Energy profiles of two types of arrangements and **d**, models of 1D β-sheet-like arrays of (*L*)-FAC18: a right-slanting arrangement (I) and a left-slanting alignment (II).

We only considered the "conformationally trapped" organization in modelling, mainly for two reasons (see also Fig. 1 in main text). First, the amide I band of the nanostructures formed by (*L*)-FAC18 in hexane centred at 1650 cm^{-1} is indicative of the incomplete hydrogen bonding between the amino acid segments.[1] Second, from the energy profiles of the 1D arrays of (*L*)-FAC18 with two types of arrangements, the β-sheet-like arrangement is indeed more energetically favourable, but a left-slanting alignment (**II** in d) is preferred for (*L*)-FAC18, in contradiction to the experimental observations (a right-slanting alignment alike **I** was observed). Furthermore, the kinetically controlled formation of supramolecular aggregates, of which the molecular building blocks are trapped by intramolecular hydrogen bonding, has been shown in many examples recently[2-4].

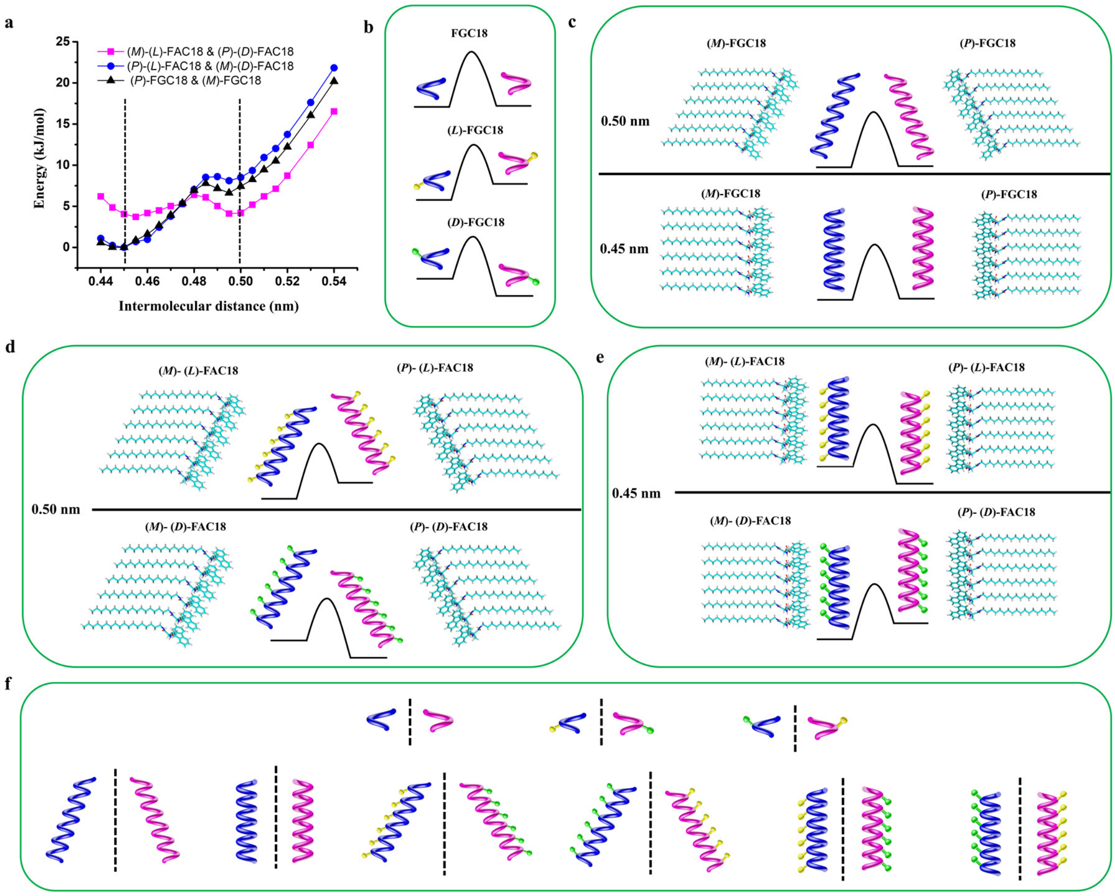

Supplementary Figure 4. The relationships between molecular chirality, helicity and 1D supramolecular organization. a, Energy profiles of FGC18, (*L*)-FAC18 and (*D*)-FAC18. The two conformers that are mirror image related have the same profile. **b**, Illustration of the energy differences between opposite twisting forms of FGC18, (*L*)-FAC18 and (*D*)-FAC18. Blue and magenta helixes represent *M*- and *P*-type twisting molecular forms. The yellow ((*L*)-FAC18) and green ((*D*)-FAC18) pendent balls represent the methyl groups at the stereogenic centres. **c**, Structural models of the arrays of (*P*)-FGC18 and (*M*)-FGC18 at intermolecular distances of 0.50 and 0.45 nm, respectively. **d**,**e**, Structural models of the arrays of (*L*)-FAC18 and (*D*)-FAC18 at intermolecular distances of 0.50 and 0.45 nm, respectively. For each organization, a row of molecules is illustrated using the "helix" representation. **f**, Summary of the mirror relationships between different molecular conformations and between supramolecular organizations.

The structural transition in arrangement, from oblique to near-rectangular, is driven by the demand for optimum spacing between the three parts of a molecule, that is, ~0.35 nm for the aromatic group, 0.47-0.53 nm for the amino acid segment and 0.43-0.44 nm for the flat lying alkyl chains.

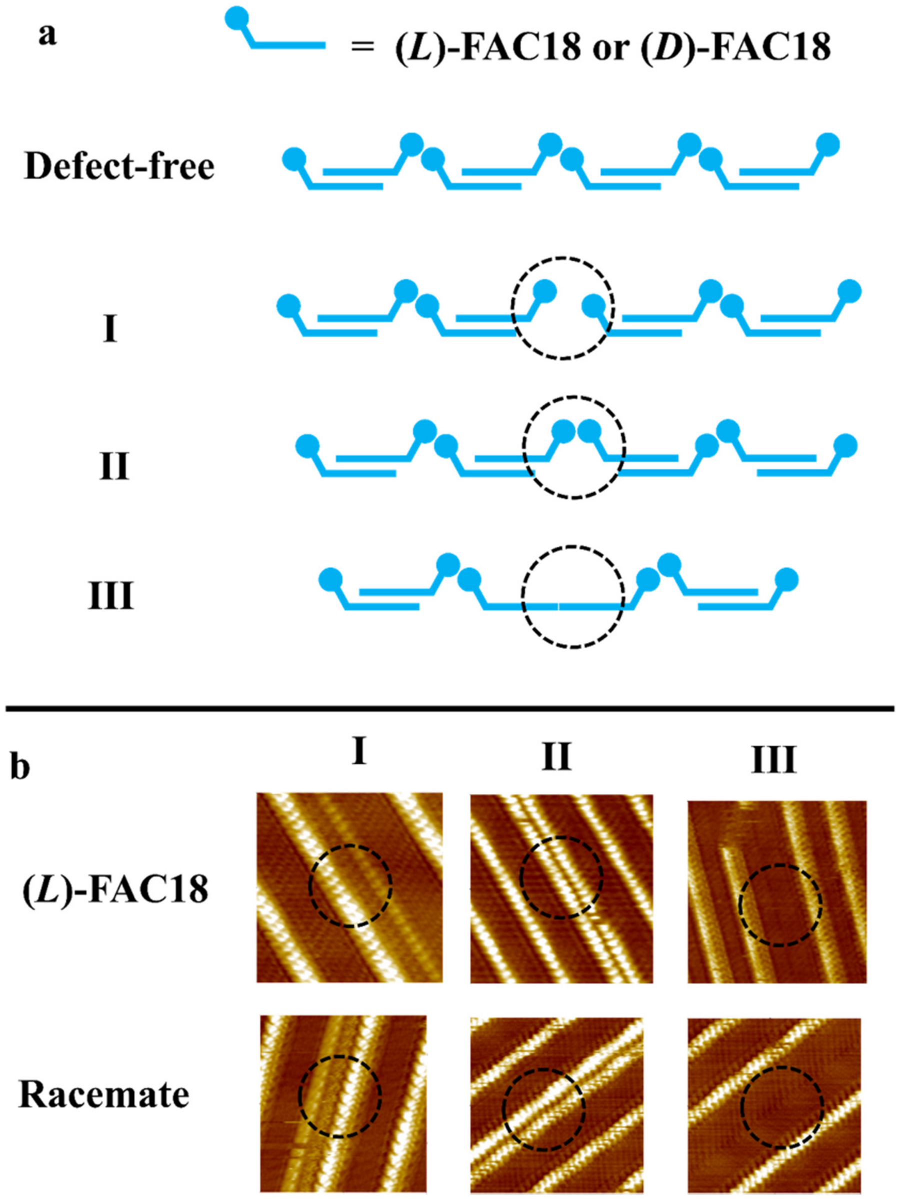

Supplementary Figure 5. Mismatch between rows of molecules. a, Schematic illustrations of the three types of defects in bilayers, **I**-**III**. **I** represents a dislocation, where the two neighbouring rows, one in the top layer and one in the bottom layer, are not connected by π-π interactions. **II** and **III**: Alkyl chains of adjacent rows are pointing in opposite directions. Such mismatching can be expected when two oppositely oriented domains meet. **b**, Experimental observations of the three types of defects in the supramolecular structures of the pure enantiomer and the racemate.

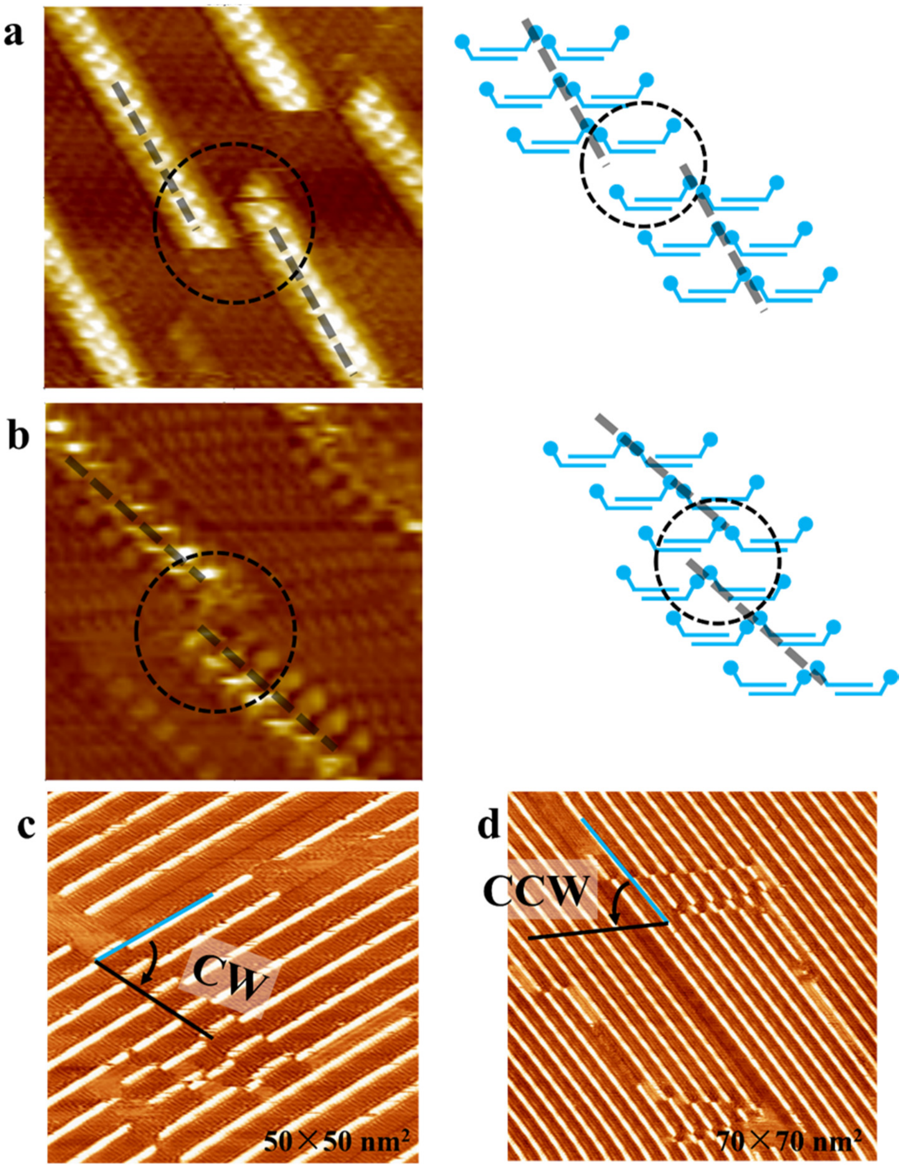

Supplementary Figure 6. Mismatch/misalignment in a row of pure enantiomers. a, STM image and schematic illustration of a dislocation in the lamellar structure of (*L*)-FAC18. The top part and the bottom part of a row run along the same direction. **b**, STM image of schematic illustration of a mismatch in the lamellar structure of (*D*)-FAC18. The top part and the bottom part of a row are oppositely oriented. **c**,**d**, STM images showing misalignments in the surface structures of (*L*)-FAC18 and (*D*)-FAC18, respectively. The connection of kink sites follows the orientation of the alkyl chains (indicated by black lines), therefore we define the misalignments in the lamellar networks of (*L*)-FAC18 and (*D*)-FAC18 as CW-type and CCW-type, respectively.

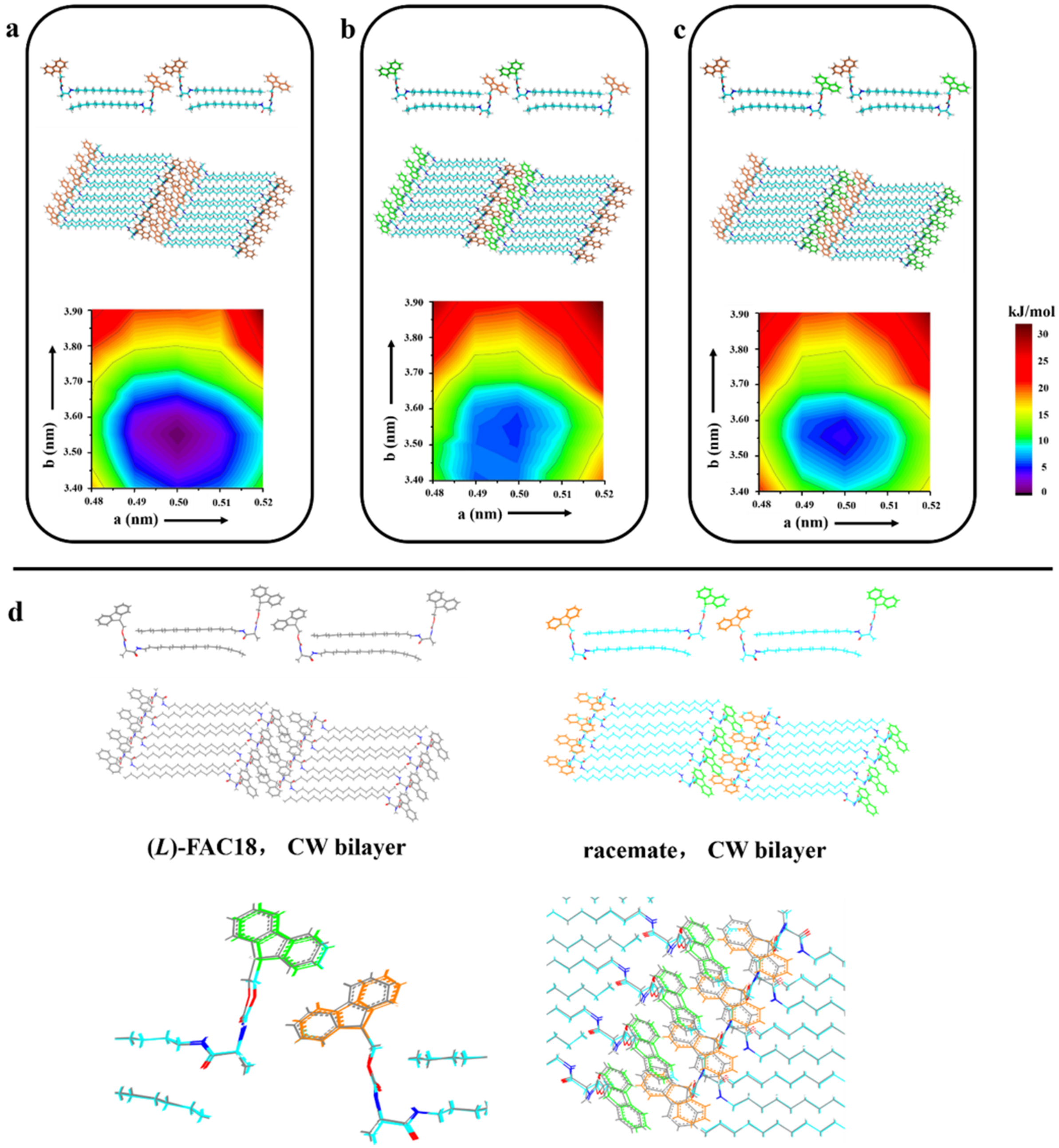

Supplementary Figure 7. Comparison between the bilayers of the pure enantiomer and the racemate. a,b,c, Side views, top views and potential energy surfaces of the MM-calculated CW structures of (*L*)-FAC18 (**a**) and the racemate **(b**,**c**). Unit cell dimensions are 0.50 × 3.55 nm^2, γ = 70°. The fluorene groups of (*L*)-FAC18 and (*D*)-FAC18 are coloured in orange and green for clarity. The global minimum of the bilayer of (*L*)-FAC18 was set to zero for comparison. **d,** The DFT optimized CW bilayers of (*L*)-FAC18 (in grey) and the racemate (green is (*D*)-FAC18 and orange is (*D*)-FAC18). Superimposed models are displayed at the bottom to show the variation in molecular conformation and arrangement.

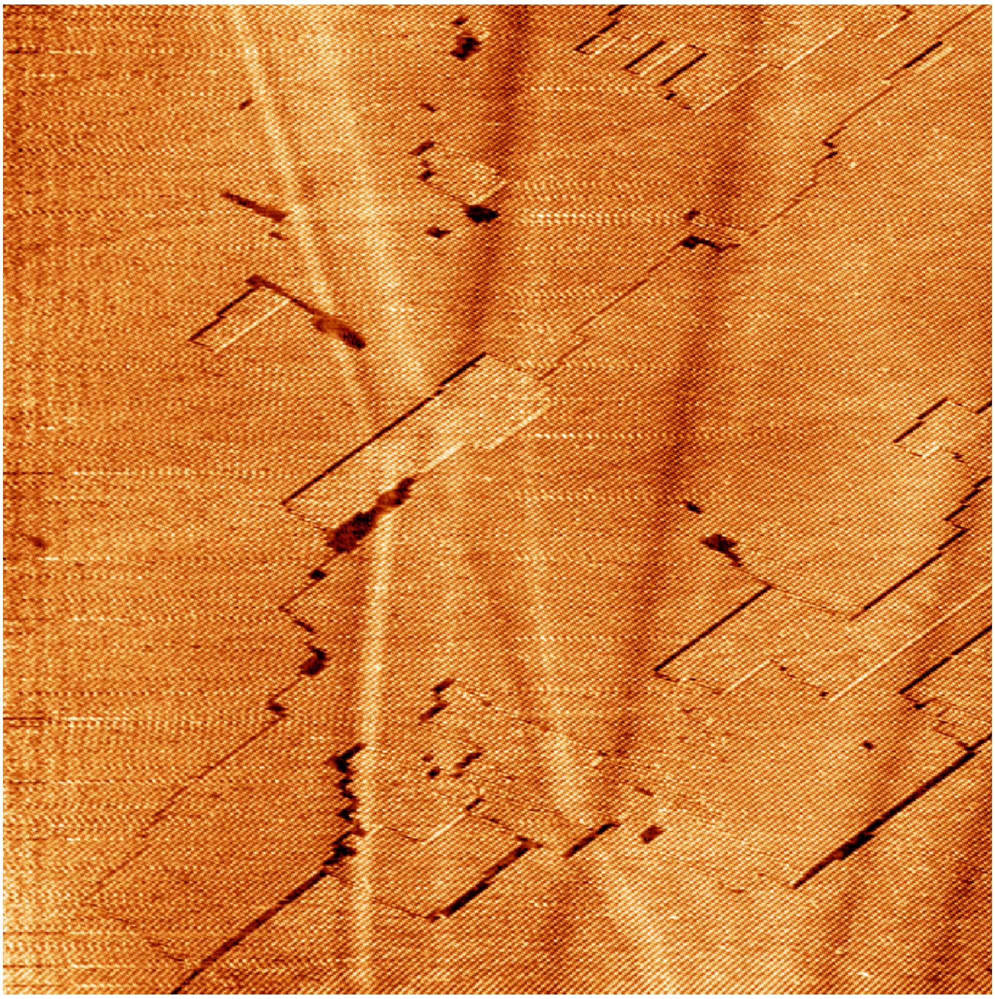

Supplementary Figure 8. Racemate. A 600 × 600 nm^2 STM image of the lamellar structures of the racemate.

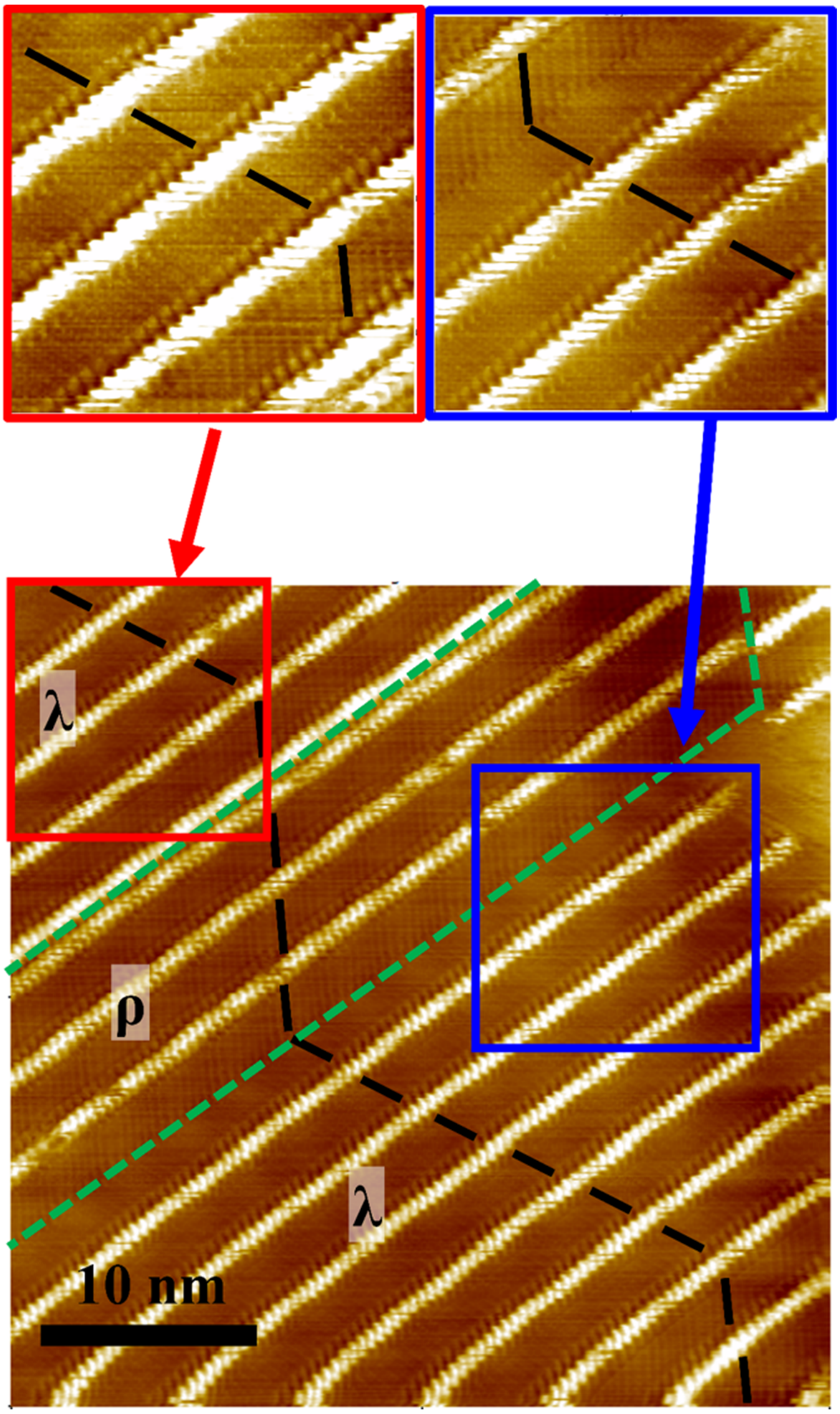

Supplementary Figure 9. Supplementary image of Figure 4c. The zigzag pattern of alkyl chains in Figure 3b was determined in different ways. The CW chirality was determined by sequential STM measurements at selected areas. The CCW chirality can be directly identified from the magnified image shown at the bottom. The orientation of alkyl chains is outlined by black lines. The boundaries between λ and ρ grains are outlined by green dashed lines.

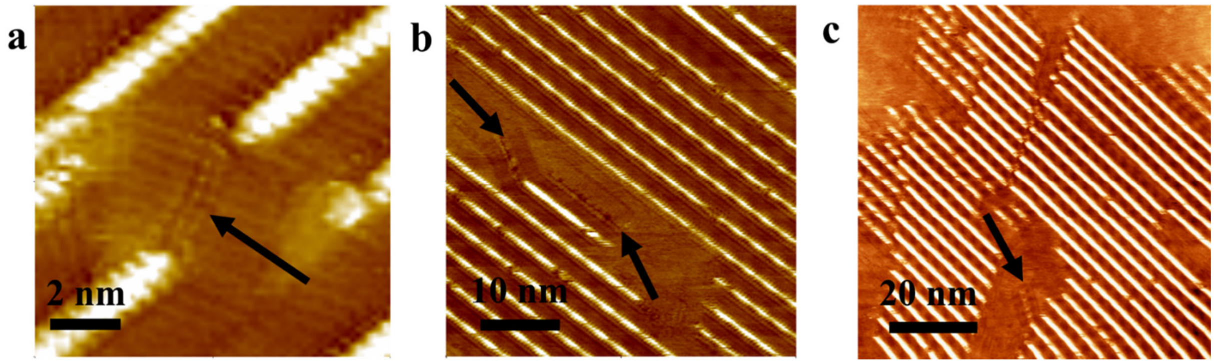

Supplementary Figure 10. Additional STM images of monolayer δ phase. a, STM image showing a small patch of δ phase. **b**,**c**, Additional STM images showing small patches of δ phase at a submonolayer coverage of the lamellar structures of the racemate. The δ phase is indicated by black arrows.

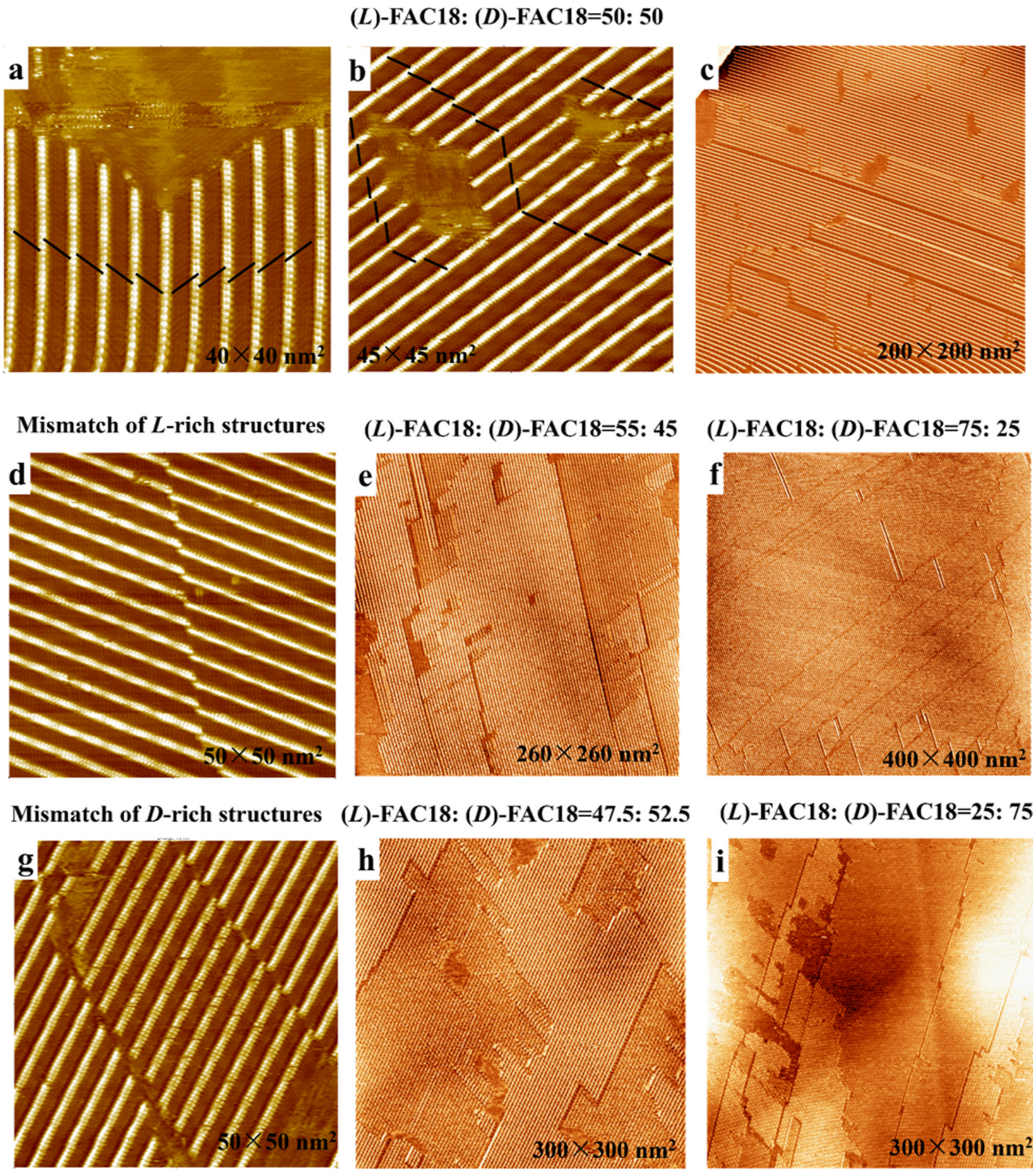

Supplementary Figure 11. Zigzag pattern of the racemate and the orientation of misalignments in non-racemic mixtures. a, b, The zigzag arrangement observed in the lamellar networks of the racemate. Black lines indicate the orientation of the alkyl chains. **c**, Large scale STM image of the racemate, where the connections of mismatches do not show a preference for a given direction. **d**,**g**, STM images showing the directional misalignments in the surface structures of the *L*-rich and *D*-rich non-racemic mixtures, respectively. **e**,**f**,**h**,**i**, Large scale STM images showing the strong preference for CW-type or CCW-type misalignments in the networks of non-racemic mixtures.

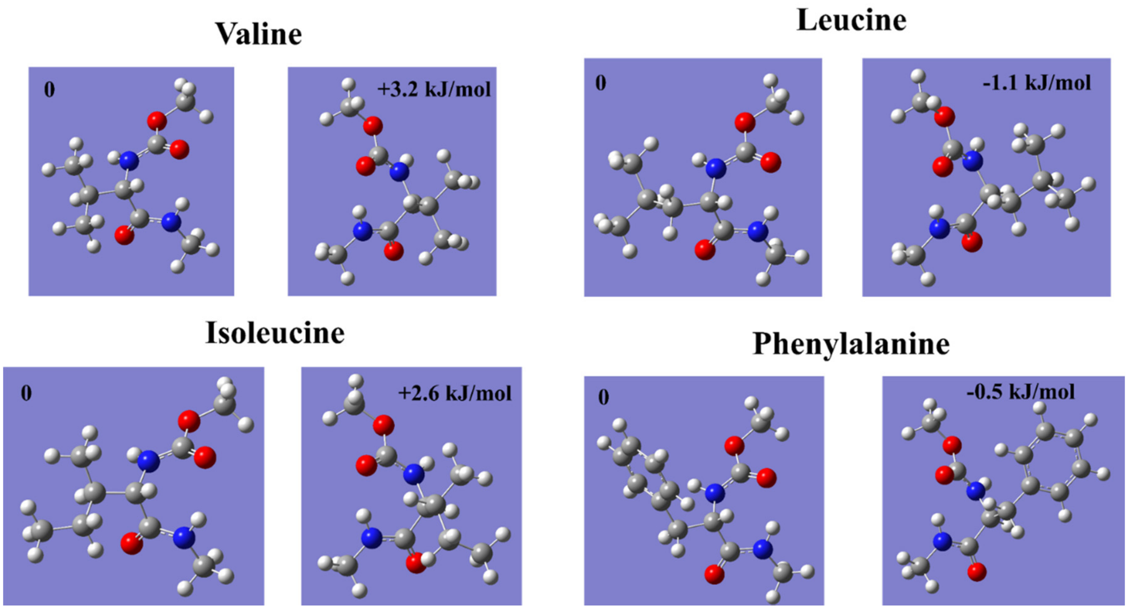

Supplementary Figure 12. Additional examples of conformational flexibility of amino acid. DFT-optimized opposite twisting forms of a few amino acids (valine, leucine, isoleucine and phenylalanine) analogues, the energy differences between two conformers of an enantiomer are indicated.

Supplementary Discussion:

Unlike deposition in vacuum via gas-phase fluxes, where the ratio of two enantiomers in the gas phase is constant and barely affected by the adsorption, the selective adsorption of an enantiomer at the liquid/solid interface – if it would occur – would certainly bring some changes to the enantiomeric excess or mass balance in the liquid phase, which can be calculated in the following way:

With the knowledge of the unit cell parameters of bilayers (0.50 × 3.55 nm^2, 70°, that is, 0.83 nm^2 per molecule), it can be easily calculated that a 1 × 1 cm^2 graphite surface is able to accommodate ~1.20 × 10^{14} molecules. The number of molecules in 10 µL FAC18 solution at the concentration of 2 × 10^{-4} M, on the other hand, amounts to ~1.20 × 10^{15}. In other words, upon depositing a solution containing both enantiomers onto the surface, 10 percent of molecules are adsorbed assuming full bilayer surface coverage while the other 90 percent remain in the liquid phase. The relationship between the enantiomeric excess in the solution before deposition (*ee*), on the surface (ee_s) and in the liquid phase (ee_l) after deposition can therefore be determined by the following equation:

$$ee_s + 9ee_l = 10ee$$

According to our STM measurements, the surface is covered with 98% CW structures at *ee* = 10%. So if the CW structure can only be formed by the *L*-enantiomer, then the ee_s and ee_l can be determined as 96% and 0.44%, respectively. That is to say, the non-racemic mixture with *ee* = 10% turns nearly racemic when a full surface coverage is reached. It takes some time to reach a full surface coverage. Therefore when it goes from a clean surface to a fully-covered one, the selective adsorption and organization of majority enantiomer would gradually decrease the ee_l, which in turn would cause a rapid decrease in the coverage of CW but a rapid increase in the coverage of CCW domains with time. It contradicts with our experimental observation that there is no significant variation in surface chirality in hours of STM measurements. In addition, the surface coverage of CW structures is 85% at *ee* = 5%, which gives ee_s = 70% but ee_l = -2.2%. That is to say, a *L*-rich non-racemic mixture turns *D*-rich upon depositing on graphite. That is not likely to occur.

In addition, kinked and stepped surface structures of metal surfaces typically serve as active sites for the adsorption – in some particular cases the enantioselective adsorption – of organic species. But often the role of step edges of HOPG in molecular adsorption and organization is not taken into consideration. Therefore selectively adsorbing of the majority enantiomer is not the likely reason for amplification of chirality on surface.

Supplementary Table 1 Statistics of the surface chirality in enantiomeric mixtures of (*L*)-FAC18 and (*D*)-FAC18.

(*L*)-FAC18:(*D*)-FAC18	Total area (nm^2)/number of images/measurement times	CCW (%)	CW (%)
25:75	93900/14/3	100	0
40:60	83300/12/2	100	0
45:55	250400/21/3	95.4±1.4	4.6±1.4
47.5:52.5	102480/21/3	82.9±3.6	17.1±3.6
50:50	114100/25/6	45.0±10.0	55.0±10.0
52.5:47.5	144850/24/2	15.4±9.2	84.6±9.2
55:45	91670/22/2	2.3±2.0	97.7±2.0
60:40	10700/29/2	0	100
75:25	165600/20/2	0	100

Note: The voids and the minor monolayer phase were not taken into account.

Supplementary methods: Within the same scan area maximum 4 images were recorded (each at every corner). After that the STM tip was moved sever millimetres away to a new location. STM images were plane corrected by using SPIP software (Image Metrology A/S). Analysis was performed by grouping images taken at different locations of the same sample and different measurement times of the same molecules. In that way approximately 4-5 images were grouped into what is called a session.

Organizational chirality was determined according to the relative orientation of alkyl chains. The alkyl chains, however, can only be well visualized at a specific state of the tip, therefore only high resolution images were selected for the statistics. STM images in which the orientation of alkyl chains can be clearly identified were collected, with different sizes ranging from 40 × 40 nm^2 to 120 × 120 nm^2. The surface area of CW and CCW structures in a STM image was calculated using the SPIP software to determine the coverage of CW ($S_{CW,ij}$) and CCW ($S_{CCW,ij}$) structures in every image (*i*) measured in every session (*j*). The weighted means $S_{CW,j}$ and $S_{CCW,j}$ in each session were calculated. After that, the coverage of CW (S_{CW}) and CCW (S_{CCW}) structures of a sample, and corresponding weighted standard deviation σ were determined.

Supplementary Table 2. A comparison of the energy difference between two conformers of an enantiomer revealed with different theoretical methods

**Method**	**(*M*)-(*L*)-FAC18**	**(*P*)-(*L*)-FAC18**
B97-D/6-31++G(d,p)	0	+8.25
*w*B97X-D/6-31++G(d,p)	0	+8.06
B3LYP/6-31G(d,p)	0	+9.28
Compass-II	0	+11.33

Note: 1) Unit of the energy difference: kJ/mol; 2) B3LYP DFT and Compass-II molecular mechanics (MM) calculations were conducted with C18 alkyl chains, while C4 alkyl chains were used for the optimization with B97-D and *w*B97X-D DFT functional.

Supplementary References

1. Fleming, S., Frederix, P. W. J. M., Sasselli, I. R., Hunt, N. T., Ulijn, R. V. & Tuttle, T. Assessing the utility of Infrared spectroscopy as a structural diagnostic tool for β-Sheets in self-assembling aromatic peptide amphiphiles. *Langmuir*, **29**, 9510–9515 (2013).

2. van der Zwaag, D., de Greef, T. F. A. & Meijer, E. W. Programmable supramolecular polymerizations. *Angew. Chem. Int. Ed*. **54**, 8334-8336 (2015).

3. Ogi, S., Stepanenko, V., Sugiyasu, K., Takeuchi, M. & Würthner, F. Mechanism of self-assembly process and seeded supramolecular polymerization of perylene bisimide organogelator. *J. Am. Chem. Soc.* **137**, 3300–3307 (2015).

4. Kang, J., Miyajima, D., Mori, T., Inoue, Y., Itoh, Y. & Aida, T. A rational strategy for the realization of chain-growth supramolecular polymerization. *Science* **347**, 646-651 (2015).
